# Bronchiectasis with secondary pulmonary infection in a child: A case report

**DOI:** 10.1097/MD.0000000000022475

**Published:** 2020-09-25

**Authors:** Ting Zhu, Haoxiang Gu, Angela Vinturache, Guodong Ding, Min Lu

**Affiliations:** a Department of Respiratory Medicine, Shanghai Children's Hospital, Shanghai Jiao Tong University, Shanghai, China; b Department of Obstetrics and Gynecology, Queen Elizabeth II Hospital, Alberta, Canada.

**Keywords:** bronchiectasis, children, pulmonary infection, wet cough

## Abstract

**Rationale:**

Although bronchiectasis is conventionally considered a chronic pulmonary disease of adulthood, knowledge of pediatric bronchiectasis not related to cystic fibrosis started to emerge. Limited information in this field is available and the management is based on expert opinion.

**Patient concerns:**

An 8-year-old girl admitted for 7 days history of wet cough, purulent fetid sputum, shortness of breath and low-grade fever. The wet cough has presented for the past 4 years, during which she had frequent hospitalization for recurrent lower respiratory tract infections.

**Diagnosis:**

Chest high-resolution computerized tomography revealed diffuse bronchial dilations accompanied by inflammation in the bilateral lung fields. Microbiologic investigation for bronchoalveolar lavage fluid was positive for *Pseudomonas aeruginosa*.

**Interventions:**

With a working diagnosis of bronchiectasis with secondary pulmonary infection, sensitive *cefoperazone-sulbactam* was administrated for 14 days with gradual improvement of clinical symptoms. Bronchoscopy washing substantially soothed the symptoms, reducing the cough and sputum volumes.

**Outcomes:**

The child was discharged after 14 days, and treated on long-term prophylactic antibiotic use (*amoxicillin-clavulanic acid*, 20 mg/kg/d, ≥ 4 weeks).

**Lessons:**

Although bronchiectasisis are condition in childhood, the diagnosis is suspected in children with persistent wet or productive cough, and should be confirmed by a chest high-resolution computerized tomography scan. Antibiotics and airway clearance techniques represent the milestones of bronchiectasis management although there are only a few guidelines in children.

## 1 Introduction

Bronchiectasis is a chronic pulmonary disease characterized by a progressive and often irreversible bronchial dilatation that presents clinically as chronic wet or productive cough accompanied by recurrent pulmonary exacerbations.^[1]^ The diagnosis is confirmed by a chest high-resolution computerized tomography (HRCT) scan. Although regarded as an orphan disease in high-income countries, bronchiectasis remains a major contributor to chronic respiratory morbidity in low- and middle-income countries.^[2]^ Moreover, delays in diagnosis of bronchiectasis of years commonly occur in children, and it is likely that many remain undiagnosed and untreated, risking premature and accelerated pulmonary decline.^[3]^ In this article we presented a case of delayed diagnosis of bronchiectasis in a child that attended respirology services with an acute event of a secondary pulmonary infection. We discuss elements of pathobiology, diagnosis, and management of our patient in the context of the present knowledge of bronchiectasis in children.

## 2 Case presentation

**Figure 1:**
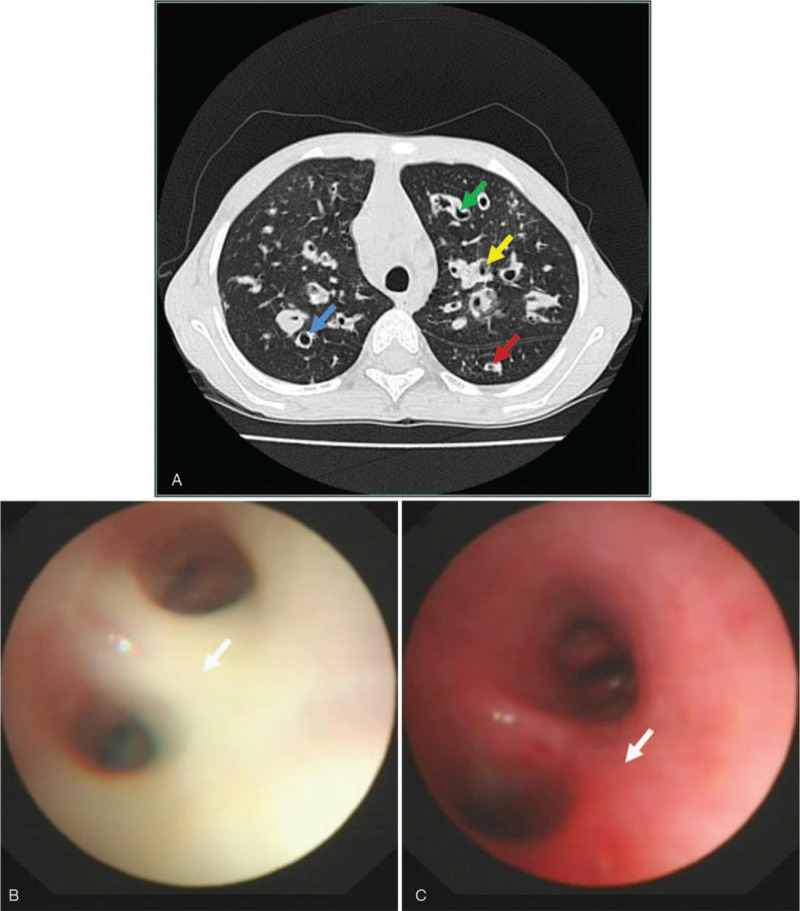
Chest high-resolution computerized tomography scan (A) and bronchoscopy (B and C) in an8-year-old girl with bronchiectasis. Dilatated bronchi and the adjacent blood vessels are visible, with the typical signet ring shape: cylindrical bronchiectasisis present (blue arrow), and varicose-to-cystic bronchiectasis is present (yellow arrow). Non-tapering of the bronchi is visible (green arrow), and visible bronchi adjacent to the mediastinal pleura or within the outer 1 to 2 cm of the lung fields are present (red arrow). Flexible bronchoscopy showed an excess amount of thick and sticky mucus and inflammatory products within the bronchial lumen (B) (white arrow), and several aliquots of sterile normal saline were instilled into the most macroscopically inflamed bronchi and suctioned immediately into a mucus trap (C) (white arrow).

An 8-year-old girl from a countryside community, Jiangsu province, Southeast China was admitted to the Department of Respiratory Medicine, Shanghai Children's Hospital, with complains of 7 days history of wet cough, purulent fetid sputum, shortness of breath, and low-grade fever (axillary temperature 37.8°C). Her mother reported that girl's symptoms initially presented at the age of 4, in the absence of obvious predisposing factors. Subsequently, recurrent respiratory exacerbations and lower respiratory tract infections occurred, requiring frequent hospitalizations in the local primary healthcare facilities of the province. The child had no other significant past medical history, no history of tuberculosis or HIV infection, and there was no family history of a similar condition. Physical examination showed chronic undernourishment [body mass index 11.3 kg/m^2^, < 3th percentile (12.7 kg/m^2^) for age and sex according to the body mass index growth curves for Chinese children and adolescents aged 0–18 years],^[4]^ nail clubbing, and diffuse fine crackles on chest auscultation. Chest HRCT revealed diffuse bronchial dilations accompanied by inflammation in the bilateral lung fields (Fig. 1: Panel A). Immune function parameters including cellular and humoral immunity were within the normal range. Spirometry test demonstrated a mixed obstructive and restrictive airway pattern (VC_max_1.03 L, FEV_1_0.73 L/s, FVC 0.99 L, FEV_1_/FVC 0.74, FEF_25–75_0.45 L/s). Flexible bronchoscopy showed an excess amount of thick and sticky mucus and inflammatory products within the bronchial lumen (Fig. 1: Panel B), and several aliquots of sterile normal saline were instilled into the most macroscopically inflamed bronchi and suctioned immediately into a mucus trap (Fig. 1: Panel C). Microbiologic investigation for bronchoalveolar lavage (BAL) fluid was positive for *Pseudomonas aeruginosa*. Genetic investigation was launched to identify the primary cause of bronchiectasis. Absence of mutations in *DNAI1*, *DNAH5*, and *CFTR* genes ruled out primary ciliary dyskinesia and cystic fibrosis (CF). With a working diagnosis of bronchiectasis with secondary pulmonary infection, sensitive *cefoperazone-sulbactam* (150 mg/kg/d) was administrated for 14 days with gradual improvement of clinical symptoms. Bronchoscopy washing substantially soothed the symptoms, reducing the cough and sputum volumes. The child was discharged on long-term prophylactic antibiotic use (*amoxicillin-clavulanic acid*, 20 mg/kg/d, ≥ 4 weeks). Immunizations including routine vaccinations and timely annual influenza and pneumococcal vaccinations according to national guidelines were recommended. Further genetic testing for alpha-1 antitrypsin deficiency and primary immunodeficiency was considered.

## 3 Discussion and conclusion

Although bronchiectasis is conventionally considered a chronic pulmonary disease of adulthood, knowledge of pediatric bronchiectasis not related to CF started to emerge. Limited information in this field is available and the management is based on expert opinion.^[2]^ Therefore, we discuss the elements of pathobiology, diagnosis, and management of our patient in the context of the present knowledge of bronchiectasis in children.

### 3.1 Epidemiology

Epidemiological information on pediatric bronchiectasis is scarce, with large variability in the incidence rate of the disease reported worldwide (0.2–735.0 cases per 1,00,000 children).^[5]^ Although the prevalence of bronchiectasis has declined significantly in most developed countries, in socially disadvantaged indigenous pediatric populations in high-income countries, and by extrapolation, for children in low- and middle-income countries, where there is overcrowding, poor hygiene, and limited access to healthcare, the prevalence remains high.^[5]^ In China, the reported prevalence of bronchiectasisis of 1200 per 1,00,000 adults >40 years of age;^[6]^ however, the prevalence in children is unknown.

### 3.2 Etiology

Pediatric bronchiectasis has multiple etiologies and is often associated with other diseases. A systematic review of non-CF bronchiectasis in children found that 63% had an identifiable underlying cause. Previous pneumonia and recurrent lower airway infections were the most common causes; others risk factors included primary immune deficiencies, primary ciliary dyskinesia, foreign body aspiration, and structural airways abnormalities.^[7]^ With the universal national immunization program and effective anti-tuberculosis therapy, the major causes of bronchiectasis have shifted from pertussis, measles, and tuberculosis to bacterial, mycoplasmal, and viral pneumonia during the past 5 decades.^[6]^ The child in our case is from a rural, economically disadvantaged background, suffering of previous pneumonia and recurrent lower airway infections. At present we cannot infer if the repeated respiratory infections were the cause of bronchiectasis in this child, but it is likely they acted as promoting, if not etiological factors in the progression of the disease. The malnutrition and possible suboptimal management of the respiratory tract infections in the primary care facilities may have contributed in the persistence of chronic respiratory changes and recurrence of symptoms.

### 3.3 Diagnosis

The clinical features of pediatric bronchiectasis are different from the adult and vary considerably, depending on age, disease severity, and clinical setting. Chronic cough is the most dominant and consistent symptom. Of note, as young children usually do not expectorate, the cough is rather wet than productive. Other clinical findings include exertional dyspnea, recurrent wheezing, digital clubbing, chest wall deformity, failure to thrive, and haemoptysis.^[2]^ In our study, the patient exhibited recurrent wet cough for several years, which was accompanied by exertional dyspnea, digital clubbing, and malnutrition. The child presented in our service, however, with what we considered to be an exacerbation of the disease. There is no validated definition of exacerbation in pediatric bronchiectasis. Exacerbation criteria used in adults (increased cough, sputum volume and worsening of purulence) are less useful in children who are often unable to expectorate. As defined elsewhere, we used the increase in cough frequency and changes in its features, and the increase in crepitations and wheezing on chest auscultation as clinical indicators of exacerbation.^[3]^

Presence of the signet ring sign (increased broncho-arterial ratio) is the pathognomonic sign of bronchiectasis on chest HRCT scans, the gold standard diagnostic tool. In children, a ratio > 0.8 is considered typical sign of bronchiectasis,^[1]^ as shown in our case. The scan showed additional radiological signs indicative of bronchiectasis: bronchial wall thickening and lack of bronchial tapering due to diffuse bronchial inflammation in both lungs, and bronchi visible close to the pleural surface.^[2,8]^ Of note, scans may appear normal due to suboptimal imaging or motion artefact.^[1]^ Therefore, bronchiectasis diagnosis should not be based purely on radiographic criteria but should be supported by relevant clinical history and findings.

Although spirometry is challenging to perform in children, it may provide useful functional information on disease severity. Spirometry can diagnose either an obstructive or mixed obstructive/restrictive airflow pattern, the child from our report showing the mixed pattern of disease.^[3]^

### 3.4 Management

Bronchiectasis requires multidisciplinary management in order to control symptoms, reduce exacerbations, and preserve lung function. Antibiotics and airway clearance techniques represent the milestones of bronchiectasis management, although there are only a few guidelines in children.

Antibiotics are prescribed to treat acute exacerbations or as prophylaxis to reduce the frequency of acute events and diminish the bacterial load and inflammation. The use and choice of antibiotics is guided by severity of exacerbations and microbiology results of the sputum culture and, whenever possible, by the BAL culture. The most commonly isolated bacteria in children are *Haemophilus influenzae*, *Streptococcus pneumonia*, and *Moraxella catarrhalis.*^[2,3]^*Pseudomonas aeruginosa* and *Staphylococcus aureus* are more often detected in older children or associated with underlying diseases with increased lung damage.^[2,3]^ In the present study, *Pseudomonas aeruginosa* isolated from the BAL culture was treated with cephalosporins with gradual improvement of symptomatology.

It is unclear which pediatric patients are more likely to benefit from long-term antibiotics and what is the optimum duration of the antibiotic treatment. A course of at least 10–14 days of parenteral antibiotic administration was recommended for children in whom the oral course fail to control the acute exacerbations.^[9]^ A recent review of 15 studies including 925 adults and children with non-CF bronchiectasis showed that a prolonged course of antibiotics (≥4 weeks) reduced the rates of exacerbations and hospitalization, although the risk of drug resistance also increased more than 3 fold.^[10]^

Despite lacking a robust evidence-based clinical efficacy, numerous airway clearance techniques are used for stable or in exacerbations of bronchiectasis.^[1,2]^ A Cochrane review concluded that airway clearance techniques appear to be safe for children and adults with stable bronchiectasis, improving sputum expectoration, lung function, and health-related quality of life.^[11]^ In our study, bronchoscopy washing soothed the symptoms, reducing the cough and sputum volumes, suggesting that BAL may be another direct and effective method to improve airway clearance and shorten the duration of illness.

Immunization and prevention of infections are integral part of the management in childhood. Measles, pertussis, seasonal influenza, Haemophilus influenzae type B, and pneumococcal vaccines should be recommended.^[9,12]^ The vaccination status was reviewed in our patient and an immunization plan was devised. Surgical intervention (segmentectomy, lobectomy) is uncommon in children. Our patient was not a candidate for this surgical management as she had no severe damage or localized bronchiectasis and responded well to medical therapy.

Although bronchiectasisis are condition during childhood, the diagnosis should be considered in children with persistent symptomatology. The diagnosis is confirmed by a chest HRCT scan. Antibiotics and airway clearance techniques represent the milestones of bronchiectasis management although there are only a few guidelines in children.

## Author contributions

**Conceptualization**: Min Lu, Guodong Ding.

**Resources**: Haoxiang Gu.

**Validation**: Haoxiang Gu.

**Supervision**: Guodong Ding, Min Lu

**Visualization**: Guodong Ding, Min Lu.

**Writing – original draft**: Ting Zhu.

**Writing – review and editing**: Angela Vinturache.
